# Automated video-mosaicking approach for confocal microscopic imaging *in vivo*: an approach to address challenges in imaging living tissue and extend field of view

**DOI:** 10.1038/s41598-017-11072-9

**Published:** 2017-09-07

**Authors:** Kivanc Kose, Mengran Gou, Oriol Yélamos, Miguel Cordova, Anthony M. Rossi, Kishwer S. Nehal, Eileen S. Flores, Octavia Camps, Jennifer G. Dy, Dana H. Brooks, Milind Rajadhyaksha

**Affiliations:** 1 Dermatology Service, Memorial Sloan Kettering Cancer Center, New York, NY USA; 2 Electrical and Computer Engineering, Northeastern University, Boston, MA USA; 3 Dermatology Department, Hospital Clínic, Universitat de Barcelona, Barcelona, Spain

## Abstract

We describe a computer vision-based mosaicking method for *in vivo* videos of reflectance confocal microscopy (RCM). RCM is a microscopic imaging technique, which enables the users to rapidly examine tissue *in vivo*. Providing resolution at cellular-level morphology, RCM imaging combined with mosaicking has shown to be highly sensitive and specific for non-invasively guiding skin cancer diagnosis. However, current RCM mosaicking techniques with existing microscopes have been limited to two﻿-dimensional sequences of individual still images, acquired in a highly controlled manner, and along a specific predefined raster path, covering a limited area. The recent advent of smaller handheld microscopes is enabling acquisition of videos, acquired in a relatively uncontrolled manner and along an ad-hoc arbitrarily free-form, non-rastered path. Mosaicking of video-images (video-mosaicking) is necessary to display large areas of tissue. Our video-mosaicking methods addresses this need. The method can handle unique challenges encountered during video capture such as motion blur artifacts due to rapid motion of the microscope over the imaged area, warping in frames due to changes in contact angle and varying resolution with depth. We present test examples of video-mosaics of melanoma and non-melanoma skin cancers, to demonstrate potential clinical utility.

## Introduction

Imaging over large areas of tissue is often necessary in clinical and research settings. However, high-resolution microscopy is limited to a small field of view in a single image acquisition. To extend the field of view, mosaicking techniques – acquiring a sequence of adjacent images and stitching or blending them together at the boundaries – have been developed^1, 2–8^. Approaches for mosaicking of a two-dimensional matrix of individual still images have been developed for bench-top work on tissue specimens *in vitro*, and *ex vivo* ^4, 9–16^. In bench-top settings, still images are typically acquired in a highly controlled manner along a specific predefined raster path. However, mosaicking for the newer, increasingly powerful and widespread implementation of video-imaging on living tissue *in vivo* is significantly more challenging. In this situation, video-images are acquired in a relatively uncontrolled manner and along an ad-hoc arbitrarily free-form, non-rastered path on living humans and animals. Approaches for mosaicking of a sequence of images from a video are called “video-mosaicking^17^”.

Three problems commonly occur when video-mosaicking *in vivo* are (i) deformations - i.e. warping or distortion in images - due to physical changes (angle, pressure) at the microscope lens-to-tissue contact, (ii) abrupt motion artifacts - i.e. “jumps” or discontinuities between consecutive frames that are caused by sudden changes in depth or topography of tissue motion and/or manual and variable operator control of the microscope during imaging, and iii) optical sectioning and resolution may vary between consecutive images due to scattering and aberrations induced by changes in depth and morphology. In the presence of these difficulties, spatially overlapping images need to be “combined” to produce a video-mosaic.

Although these challenges, and our solutions presented in this paper are generic for any setting in which large field of view video-mosaics are needed from deep, high-resolution microscopic imaging in living tissue, we are, in this paper, specifically focused on the use of video-mosaicking for reflectance confocal microscopy (RCM) imaging in human skin *in vivo*. RCM imaging non-invasively acquires nuclear- and cellular-level detail with thin optical sectioning (1 – 3 *μm*) and high resolution (0.5 – 1 *μm*)^18, 19^,

Several clinical studies have consistently reported RCM imaging to be capable of diagnosing melanocytic and non-melanocytic cancers with high sensitivity and specificity^19–22^. Recently, the Centers for Medicare and Medicaid Service in the USA granted current procedural terminology (CPT) codes, which means that RCM imaging is now a billable and reimbursable procedure^23^. The imaging is advancing toward routine implementation for guiding diagnosis as it has shown to increase diagnostic accuracy while maintaining a good specificity (and reducing the rate of biopsies of benign lesions per detected malignancy)^24–28^. Meanwhile, with the advent of a new generation of handheld confocal microscopes, acquisition of videos instead of only still images is becoming increasingly useful^29–31^ to consistently and rapidly display tumor morphology and surrounding normal tissue over much larger areas of skin (from 2 *mm* × 2 *mm* up to 10 *mm* × 10 *mm*).

In such settings, the special challenges are that the microscope is manually controlled and focused at varying depths (50 to 200 *μm*) in living tissue, resulting in varying resolution, exaggerated motion and distortion between images in any video. Moreover, the regions to be imaged differ greatly from subject to subject, skin site to skin site, and lesion to lesion, so operator flexibility in acquisition is necessary. Although “playback” of videos can be useful, the ability to spatially “map” the imaged region and display the entire field of view at once, by constructing a mosaic, allows much more “natural” analysis by trained clinicians or (semi-)automated algorithms^29^. Reading and interpreting a mosaic more closely mimics the standard procedure for examining pathology slides, which usually starts by looking at 10 mm × 10 mm areas of a tissue section with 2× magnification. Thus, there is a need in the clinic for video-mosaicking of RCM images, toward wider acceptance and adoption for routine use.

In this paper, we present a new algorithm for video-mosaicking of subsurface confocal microscopic images, which addresses all of the expected challenges. Earlier video-mosaicking techniques, for both confocal and non-confocal endoscopy, either address only a subset of these problems, or attempt to avoid some of them by limiting the freedom of motion during acquisition^1–3^. Video-mosaicking was demonstrated for imaging of oral and cervical tissue^1^. However, the imaging depth was close to the surface of tissue, so tilt and loss of resolution were not significant. Similarly, another algorithm was reported on human skin and mouse brain tissue^2^, but, again, no attempt was made to handle the warp or deformation that can happen when imaging at greater depth^2^. In another study on human and mouse colon tissue^3^, the algorithm models both rigid and non-rigid deformations in a linear fashion. However, the algorithm does not consider abrupt motion artifacts between frames and, furthermore, can enhance blurring at image boundaries through the use of blending to produce the final mosaic.

We address all these challenges by (i) directly modeling deformations, (ii) automatically detecting abrupt motion artifacts (discontinuities) in the image sequence, and (iii) robustly stitching the registered frames with an approach that combines overlapped images flexibly, in a data-driven fashion, without blurring or loss of resolution. Our algorithm is based in part on adaptation of methods from computational photography^32^. In particular, we describe the use of keypoint matching to design affine transformations to allow warping among consecutive video frames (images) during registration. The properties of the resulting affine transformations are also used to detect abrupt motion artifacts between frames. Finally, we developed an approach, based on a graph-cuts based model^33^, to stitch overlapping frames together along arbitrary (non-rectilinear) boundaries, driven by the image data itself, without the loss of resolution that, otherwise, would occur from blurring or other blending techniques, and without imposing arbitrary pre-defined stitching boundaries. Herein, we describe our method in detail, and then present illustrative video-mosaicking results on human skin lesions *in vivo*. The advantages and limitations of our algorithm are discussed, as well as the possible future directions for both research and clinical applications.

## Results

Video-mosaicking is of particular interest for rapid assessment of skin lesions that can be arbitrarily large and/or located at curved, irregular anatomical sites. We illustrate the capabilities of our approach by presenting results for three distinct clinical cases, each of which presents a particular set of imaging requirements. The flexibility and robustness of our approach is demonstrated for producing useful video-mosaics in all three settings.The first case is a lentigo maligna melanoma (LMM), where the need for accurate planning of surgical margins at non-flat corrugated and irregular anatomical sites is met by video-mosaicking over arbitrarily large predefined areas of tissue. In the second case, we show how video-mosaicing can help in adaptively detecting small and focal sites of suspicion within widely spread areas for diagnosing and monitoring of extramammary Paget disease. In the third case, we illustrate how video-mosaicking can help in rapid intraoperative detection of residual basal cell carcinoma in surgical wounds. All the videos acquired for this study were obtained after Institutional Review Board approval.

### Case 1: Lentigo Maligna Melanoma (LMM)

**Figure 1:**
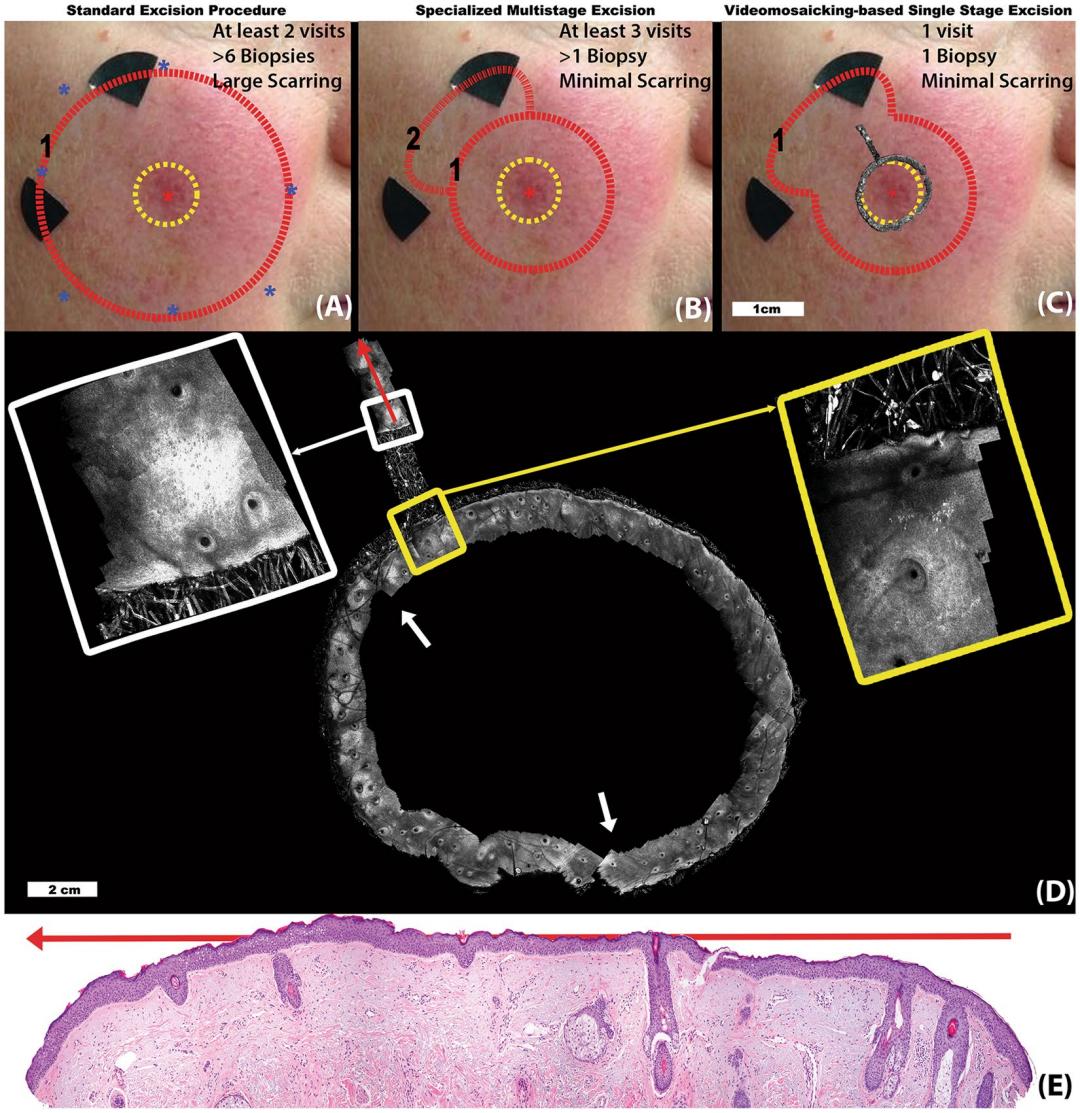
Lentigo Maligna Melanoma (LMM): Figure illustrates three potential methods to treat an LMM lesion in the clinical setting and how RCM video-mosaicking can improve LMM management. In all three approaches, following an initial diagnostic biopsy (red asterisk), the clinical margin (yellow dotted circle in (**A**–**C**)) is determined through macro imaging (dermoscopy and Wood’s lamp examination). (**A**) In the standard surgical procedure, completely excising cancer in least number of surgery is the primary concern and tissue conservation secondary. After diagnosis, in a single visit, the lesion is excised with a 1 cm wide surgical margin around the clinical margin. (**B**) At specialized cancer care clinics, in order to spare healthy tissue, multi-staged excision may be performed. In the case illustrated here, first an excision with a conservative margin (5 mm, red circle 1) was conducted. Histological examination of the excised tissue, indicated residual tumor at 11 o’clock spreading in a radial direction. Thus, after a few days, a second excision was planned and executed in that region only (red circle 2). In (**C**) we show how RCM video-mosaicking can streamline and improve the procedure. The video-mosaic of the clinical margin was created from *in vivo* RCM videos (shown overlaid on the clinical image) by the algorithm described here. Expert examination of the video-mosaic revealed tumor extension at the 11 o’clock location and extending radially (yellow inset), therefore, we extended the video-mosaic in that direction (the radial mosaic in the direction of the red arrow, white inset), all in one visit. Histopathological examination along the same line as the extended mosaic (red arrows in panels (**D**) and (**E**)) confirmed the findings in the RCM video-mosaic, showing the extent and margin of tumor cell spread towards the periphery of the lesion.

In Fig. 1, we illustrate the use of our approach for pre-surgical planning of LMM excisions. LMMs frequently occur on the face, head. and neck. The lesions usually appear to be small on the surface (millimeters in extent), but the subclinical, subsurface spread is often substantially larger (centimeters), and is also often obscured within surrounding degraded photo-aged skin. Moreover, the subsurface spread of melanoma cells is typically highly dendritic, like the roots of a tree, and therefore the thin tendrils that define the outermost margins are difficult to determine using current approaches, namely visual (clinical, dermoscopic) inspection and Wood’s lamp (ultraviolet-excited fluorescence) examination.

Following an initial diagnostic biopsy (at the location of the red asterisk at the center of panels (A), (B), and (C)), the clinical margin (yellow dotted circle) of the lesion is determined with dermoscopy and Wood’s lamp examination. In the standard surgical procedure, removal of the disease is the primary concern and tissue conservation the secondary one. Therefore, as shown in Fig. 1A, a wide “safety” margin (red dotted circle), up to 1 cm, beyond the clinical margin is estimated. The estimated margin is interrogated by blindly sampling the margin via “mapping” biopsies (blue asterisks)^34^. Several days later, if all biopsy reports result in negative findings at the margin, the lesion is excised along the surgical margins. In case any of the biopsies turn out to be positive, the surgical margin is extended further along that direction. After the surgery, if residual tumor is found at the edges of the excised tissue, then further surgery is performed on a third visit.

In specialized cancer care centers, in order to conserve more of the healthy tissue, alternative multi-stage excision guided by histology is sometimes performed (Fig. 1B). After the initial diagnostic biopsy, the central part of the lesion corresponding to the clinical margin (yellow dotted circle) plus an initial narrower “safety” margin (usually 5 mm) is excised (red dotted circle). After histopathological analysis of the excised tissue, if it turns out that the excision margin in any quadrant contains residual tumor, the patient undergoes further excision in those positive quadrant(s) (red dotted curve) and the excised tissue is processed again by pathology. This process iterates until tumor-free margins are achieved and can take several days to several weeks, depending on the size and irregularity of the tumor.

In contrast, our video-mosaicking method offers a streamlined diagnostic and therapeutic procedure, as illustrated in Fig. 1C. Subsurface structures are visualized non-invasively along the clinical margin (yellow dashed circle), as shown in the circular video-mosaic superimposed in gray-scale at the clinical margin. Assessment of the video-mosaic revealed tumor in one quadrant (yellow inset) and consequently a localized radial scan was performed next (illustrated in detail in Fig. 1D). Expanded views of the yellow and white insets show two frames along this radial scan. Analysis of the radial mosaic correctly determined the extent of the tumor growth (without the need for mapping biopsies) in the associated quadrant. of the tissue to guide further surgery. Immediately after imaging, the lesion was excised with an appropriate “safety” margin (red dotted lines). Note that the capability of RCM for subsurface imaging with cellular resolution allows non-invasive detection of the extent of disease, which otherwise is not possible without invasive biopsy.

Histopathology from this particular case confirmed the margin determined in the video-mosaic. Note that he white arrows in panel D show two locations at which our tailored video-mosaicking algorithm automatically compensates for ‘‘jumps’’ (discontinuities) in the video, allowing correct mosaicking of the three distinct arcs. The individual sub-mosaics are put together manually in an image editor. This is a simple procedure as the first and last frame of consecutive sub-mosaics are spatially located next to (or very close to each other), and their relative location to each other can typically be estimated using the RCM video.

The resulting video-mosaicking method allows sufficiently precise determination of the appropriate surgical margin^35^. The excision is tissue-conserving, similar to that of the staged excision approach used in specialized clinics, but all accomplished in a single visit without the need for repeated visits and repeated biopsies.

### Case 2: Extramammary Paget disease (EMPD)

**Figure 2:**
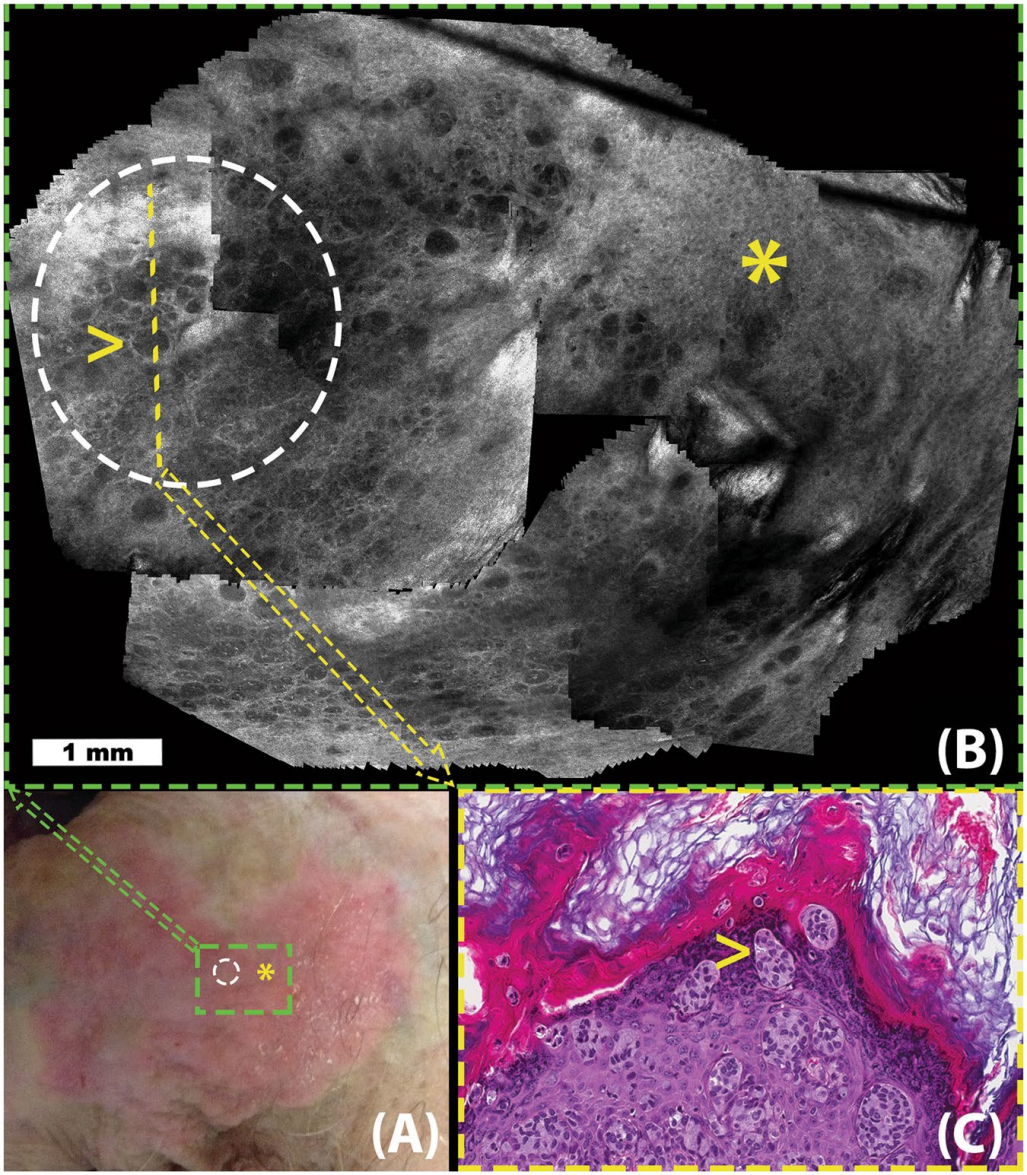
Extramammary Paget Disease (EMPD): Figure illustrates the use of video-mosaicking for non-invasive examination of tumor spread in a suspected case of EMPD. Panel A (lower left) shows a clinical image exhibiting the suspicious lesion, whose red color and appearance is suggestive of EMPD. In panel B (top), a video-mosaic is obtained by imaging with a handheld confocal microscope in a spiral fashion intended to largely and efficiently cover the green box in panel A. The region inside the white circle on panels A and B was determined to be suspicious in the video-mosaic, and thus a histological section (yellow line in panel B) was excised (shown in panel C). Histological examination of the slice confirmed the findings from the video-mosaic, as illustrated by the high density of tumor cells visible at the yellow arrow in both panels B and C. Unfortunately, during such imaging along a spiral path, the operator could not keep the imaging depth constant and the resulting artifact, as seen in upper left side of the mosaic, occurred at the overlap area. Although the algorithm stitching artifacts through grapcuts based method, such artifacts cannot be completely avoided due to the nature of RCM imaging and challenges in image capture.

In Fig. 2, we illustrate the use of video-mosaicking to improve the sampling accuracy and guide diagnosis of extramammary Paget disease (EMPD). EMPD is a rare skin cancer that appears on the genitalia, often covering large areas, and that is frequently mistaken for an inflammatory or infectious condition. In addition, due to a patchy and focal distribution of tumor cells, sampling the lesion for diagnosis and management requires numerous biopsies, most of which typically turn out to be benign.

In order to cover such large areas with curved surface topography, clinicians move the confocal microscope over the region of interest in a free-hand fashion. The approach is to acquire RCM videos in the suspicious area, starting from the center of the lesion and navigating towards the outer borders in a spiral path, aiming to cover the entire area in a rapid fashion without missing any regions. This approach allows identification of areas with higher tumor cell density, which can then be selectively biopsied to confirm diagnosis^36^.

Being able to image in a user-determined irregular trajectory is important in EMPD as tumor cells are sparsely distributed in a patchy fashion. To illustrate, in Fig. 2, we indicate a region (yellow asterisk on panels A and B) that is well within the clinical lesion but determined in the video-mosaic to be free of tumor cells. Furthermore, we also illustrate a positive area (white dotted circle in A and B), where video-mosaic suggests signs of EMPD in a clinically suspected area. The findings from video-mosaicking are also confirmed by histological examination (Fig. 2C) of the biopsied sample from the region.

### Case 3: Basal Cell Carcinomas

**Figure 3:**
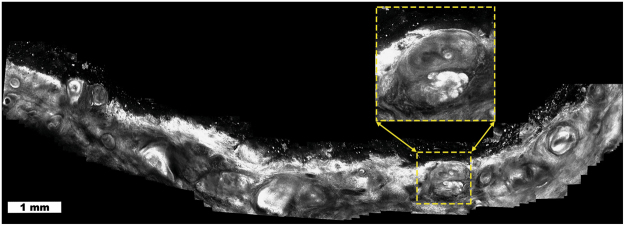
Basal cell carcinoma (BCC): Video-mosaic showing residual tumor (inset, bright lobular nests) in a quadrant of a Mohs surgical wound. Video-mosaicking enables the localization of the residual tumor by providing architectural information that is not readily available when viewing the original videos.

Fig. 3 illustrates the use of video-mosaicking for the intraoperative assessment of residual basal cell carcinoma (BCC) margins during surgery. BCC is often treated with a tissue conserving procedure called Mohs micrographic surgery, in which the surgeon is guided by the preparation and reading of frozen pathology of thin excisions, taken in successive stages. This is a relatively slow procedure since each stage takes 20–45 minutes to be processed for frozen sections and two or more stages are often required for clearance.

Video-mosaicking performed directly in the Mohs surgical wound allows rapid detection of residual tumor^30^. This enhances the ease of correlating microscopic cellular detail with macroscopic location in the wound, which would be much more difficult with standard RCM imaging or by simply observing the original videos. Since the video-mosaic has the same resolution as in the original images, the surgeon can zoom in on suspicious areas and examine them in detail, with higher magnification, without loss of any information (e.g., inset in Fig. 3).

## Discussion

In this paper, we present a new way of imaging large areas of tissue at microscopic resolution, even when the anatomical sites of interest are hard to access and/or present uneven, corrugated and irregular topology. To image such large and challenging regions of interest, clinicians must often rely on video-microscopy. Typically, a video sequence is captured over the area of interest by moving a handheld microscope manually over the tissue. The need to maintain cellular resolution means that each frame of these videos has a small field of view, and thus the video presents a rapid succession of small correlated areas of tissue but viewing them as a video does not reveal their spatial relationship. As an example of possible consequences, in the case of RCM imaging of skin, a recent study^37^ showed that diagnostic capabilities decrease slightly when the handheld version of the microscope is used. The authors attributed this decrease to the lack of mosaicking capability of the handheld microscope, and inability to quickly examine large field of view (large area of tissue). Video-mosaicking thus solves this problem by registering, concatenating, and stitching together microscopic images to produce a large field of view.

However *in vivo* microscopic video-mosaicking is challenging for both image capture and mosaic composition. The quality of the final mosaic is sensitive to the motion of the microscope over the imaged area. The operator must to avoid rapid motion, while maintaining smooth translation across the skin and constant depth of the focal plane, with micron-level accuracy. Rapid motion can result in blurring and warping deformations in the final mosaic, while rapid change in depth can result in jumps in the imaging plane between frames. Although such artifacts can not be totally avoided during clinical imaging, one of the novelties of our approach, the scene cut detection step, can detect and handle both types of rapid motion and thus minimize these artifacts in the mosaic.

Another novelty that distinguishes other video-mosaicking methods in the literature is our graph-cuts based seamless stitching approach. This method has previously been used in computational photography; however, as far as we know, this is the first study that utilizes it in the microscopy setting. The challenge it solves is that traditional blending methods will lead to blur, and thus loss of the cellular resolution that is critical to diagnosis, while image-to-image variability and edge artifacts in the images make an a priori fixed cut-and-stitch approach suboptimal. Using graph-cuts based stitching, we allow the data itself to drive the merging of adjacent overlapping images, thus preserving the resolution of the original images without altering pixel values, while providing maximally smooth transitions, across stitching seams.

However, one current challenge of video-mosaicking is processing delay. Our current implementation stitches a 500 frame video (where each frame is 1000 pixels on a side, for a total of of 500 megapixels) into a video-mosaic in less than 30 minutes, using a MATLAB based implementation with a few C MEX libraries. The timing varies depending on the specifics of the scene geometry, which affects the number of reliable keypoint matches that are used in the registration step. This is adequate for some clinical settings and indeed is being actively validated by our clinicians, as in the examples shown in the Results section. However this delay is clearly an impediment to wider adoption; being able to present video-mosaics on-the-fly to the clinician would provide valuable live visual feedback, which would not only make it more clinically useful but would also help decrease motion artifacts and ensure appropriate coverage of the region of interest. Currently, the registration step is the bottleneck to increasing the speed of video-mosaic composition. We note that our MATLAB code is not optimized for such processing, and thus we are confident that significant increase in speed is feasible without significant loss of performance. Highly efficient codes have already been developed in C and C++ that provide real time feature extraction and tracking capabilities, such as in the OpenCV computer vision library^38, 39^. In addition, all the registration steps, including the SIFT keypoint extraction, matching, and RANSAC-based keypoint elimination steps, are parallelizable and could also be implemented on GPUs for further acceleration. Therefore, we believe that substantial increase in computational speed will be achieved, enabling stitching that essentially keeps up with the image acquisition.

Another approach to lessen motion artifacts is the potential availability of smaller and faster microscopes with frame rates higher than the current 7 frames per second. Besides, with increase in processing speed, smaller microscopes would allow enhanced operator control of free-hand movement and, along with higher frame rates, further facilitate real-time stitching, thus improving both the quality of the mosaics as well as the ability to rapidly and reliably cover an entire arbitrarily shaped region of interest. Availability of real-time video-mosaicking has the potential to enable the clinicians to be more time/cost efficient and precise in their diagnostic assessments and surgical practice.

Finally, as shown by the clinical examples in the results section, video-mosaicking has the potential to open new alleys in imaging based diagnosis and surgery, that in the past the clinicians were not able to do due to technical limitations. Moreover, the initial clinical success in imaging skin, is being translated to other setting and applications. One example is video-microscopy in the oral cavity. Head and neck surgery group in our institute is currently testing handheld microscope with a new telescopic probe for intra-oral imaging during head-neck surgery. Testing has thus far been performed on then patients, with video-mosaic being assessed for correlation to pathology for delineation of margins. Again real-time mosaicing has the potential to provide live feedback to the surgeon and make surgical planning and procedure more efficient.

Need for video-mosaicking has already raised in other fields such as video-microscopy of oral cavity, where confocal microscopy has also begun to show its usability. Head and neck group in our institute is currently testing a new telescopic attachment on the handheld microscope, which was designed by our group, to acquire video-microscopy videos. Their investigate the potential use of video-microscopy in image guided surgery of the oral cancer patients. The videos that they acquire during in the OR are converted into video-mosaics for diagnostic correlation studies. Again, real-time video-mosaicking has the provide critical feedback to the surgeon throughout the surgery and make surgical planing and procedure more efficient.

## Methods

### Ethics Statement

Prior to enrollment to the study and RCM imaging, all patients signed an informed consent to an institutional review board (IRB) approved protocol at Memorial Sloan Kettering Cancer Center. The study and the experiments were conducted in accordance with the MSKCC IRB regulations and the Declaration of Helsinki principles.

### Algorithm Overview

**Figure 4:**
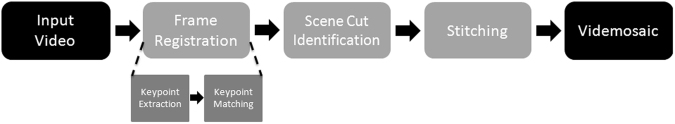
Flowchart of video-mosaicking process. The individual steps are further described in the methods section, with further details in the supplementary material.

The algorithm is composed of a series of steps (Fig. 4) designed to handle each of the three challenges listed in the introduction. We started with a classical image mosaicking pipeline from the multiview 3D reconstruction literature^32^ and added methods to *identify discontinuities* (called “scene cuts”) between frames, requiring separation into individual “subvideos” and to *stitch* with maximal preservation of image fidelity, both specifically tailored for application to video-microscopy.

**Figure 5:**
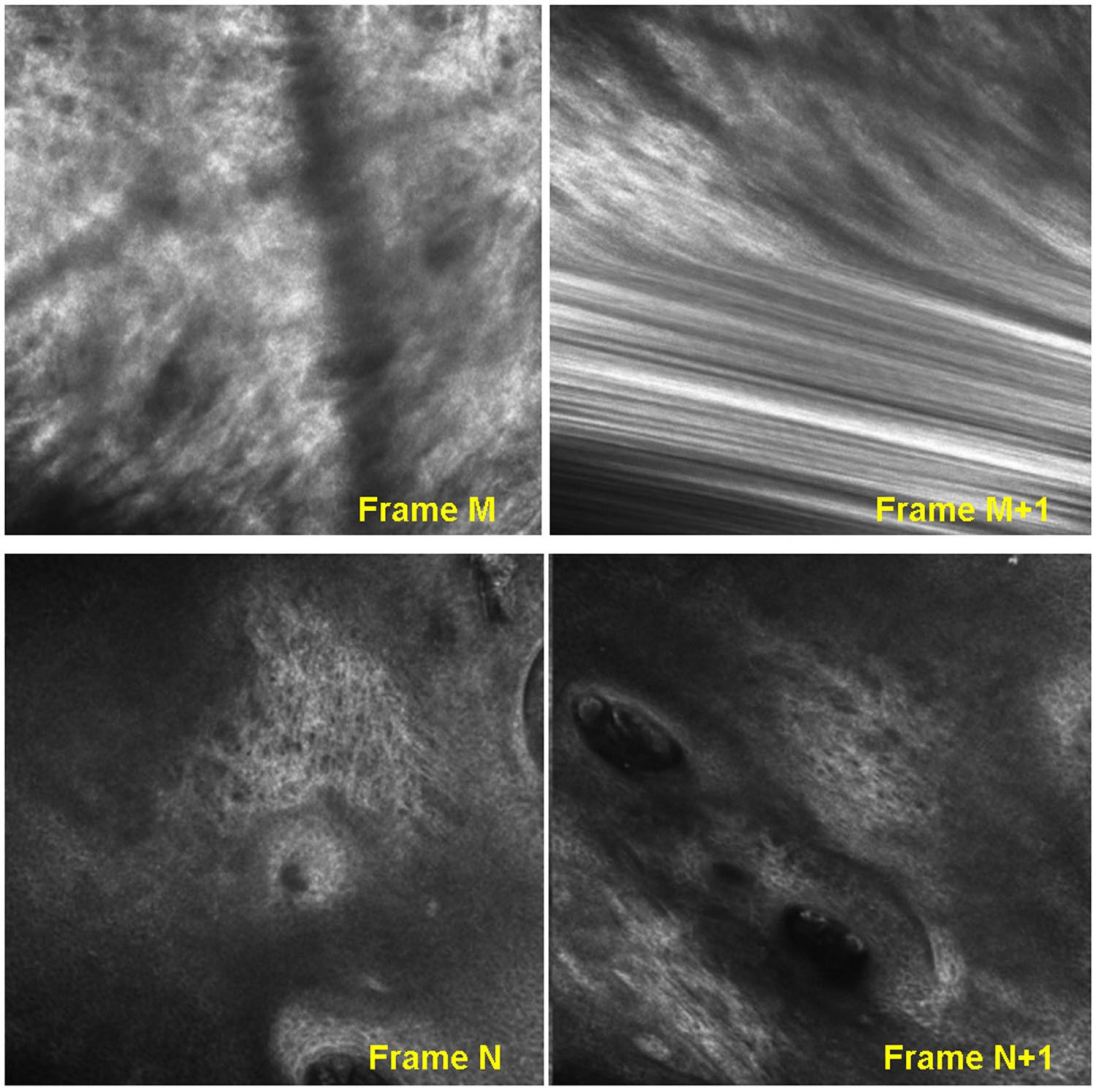
Common problems in imaging that will lead to discontinuities or “scene-cuts” in the RCM video-mosaics; (i) Blur due to rapid motion (**Row-1**), (ii) Lack of overlap between frames (**Row-2**).

Overall, the algorithm takes an RCM video as an input, registers the consecutive small FOV frames, detects any rapid motions indicating the need for scene cuts, and finally stitches all frames into a large FOV mosaic, automatically within subvideos and automatically or semi-automatically across scene cuts (Fig. 4). The registration procedure itself is composed of two subroutines, keypoint extraction^32, 40^ and keypoint matching^32^. During *keypoint extraction*, uniquely identifiable pixel locations in each frame are determined. Then, through *keypoint matching*, keypoints are matched between consecutive frames and their inter-frame displacement is tracked. These displacement parameters are then used to estimate registration parameters to map the pixels of each successive frame to the reference frame of the mosaic. These registration parameters also provide quantitative estimates of inter-frame motion. Therefore, the algorithm uses them to identify rapid and/or tilted motion in the videos where inter-frame registration is either not feasible or unreliable; at those frames the algorithm automatically imposes scene cuts (Fig. 5). Each resulting contiguous segment is stitched into an individual video-mosaic. Since consecutive frames overlap significantly, rather than using common algorithms based on blending, which blur details and coarsen resolution, we adopted a graph-cuts based stitching algorithm^41^ which retains the original values for each pixel in the mosaic, critical to preserve cellular detail. In the following sections, we briefly describe each of these steps; details are in the supplementary material.

### Frame Registration

Since the user moves the microscope freely over the region of interest without any positioning feedback, motion information must be extracted by analyzing the image frames themselves. We use a two step approach (Fig. 4) to estimate the microscope motion information: (i) keypoint extraction, and (ii) keypoint matching.

#### Keypoint extraction

We use an optical flow estimator that tracks the inter-frame motion of uniquely identifiable spatial locations, referred as “keypoints”. In macroscopic images of the natural world images, keypoints are usually found at high-contrast locations such as corners and local extrema. Here they are not as clearly identifiable, which necessitates some further processing as described below. Keypoints are detected by their relation with their neighbors, and matched between consecutive frames using their descriptors.

Due to changes in microscope-to-skin contact parameters (angle, pressure, velocity) during video capture, the scale, orientation, and intensity value of keypoints may change slightly between consecutive frames. To improve robustness, we use “Scale-Invariant Feature Transform” (*SIFT*)^40^ keypoints and descriptors, among several available descriptors in the literature^32^. SIFT is invariant to commonly encountered variation in images such as uniform scaling and changes in orientation, and also robust against affine distortion and changes in intensity. The first step in the SIFT algorithm is keypoint identification. Keypoints are typically high contrast pixels within their neighbors. SIFT uses a difference-of-Gaussian (DoG) representation (Sup. Fig. S1) At the end of this keypoint extraction stage, we typically acquire ∼3000 keypoints per frame. Each keypoint is composed of two components, a spatial location (coming from the extrema detection step) and a 128-length descriptor vector (histogram representing the distribution of the gradients coming from the SIFT algorithm)﻿﻿ (Sup. Fig. S2).

#### Keypoint Matching

In order to estimate a geometric transformation between frames, corresponding keypoints must be reliably matched despite varying intensity and scale. The keypoint descriptor used by SIFT is robust to such changes because it relies on the distribution of the gradients (histogram) in the local neighborhood of the keypoint; moreover we normalize orientation using the direction of the maximum gradient^40^ in the keypoint’s neighborhood to provide invariance against rotational motion.

We used the two step keypoint matching procedure described in ref. 32 to estimate displacement between consecutive frames. Two requirements are considered during the matching procedure: (i) descriptors of the keypoints should be similar, and (ii) relative displacement of the matched keypoints should be similar. The second condition follows from the fact that all keypoints should move in a tandem fashion, consistent with translation, rotation, and change of (projection) angle of the microscope, but not a general non-rigid morphing. Specifically, the global displacement of the keypoints should be well approximated using a single registration transformation.

Since the keypoint estimator itself is noisy with many outliers, we systematically eliminate unreliable matches that do not comply with this restricted type of motion. All keypoints in a subsequent frame are provisionally matched with the two “most similar” keypoints in the previous frame as determined by the *ℓ*_2_ (Euclidean) distances between their SIFT descriptors, which we use as our keypoint similarity metric. A keypoint is then considered non-uniquely matched, and therefore the match is designated as unreliable, if the two most similar keypoints in the previous frame are nearly equally similar to the given keypoint, as measured by their similarity metric ratio being below a threshold (heuristically set in our experiments to 1.2). For example, a keypoint located in a region with similar or repeating textures is typically not unique. We prune our set of keypoint matches by discarding matches deemed unreliable by this procedure.

Next, the pruned matches are used to model the transformation that best registers consecutive frames. In principle, only 3 keypoint matches are needed to determine the three registration parameters, and those parameters should be consistent across the choice of any three keypoints. However, in practice there will still be outlier correspondences, even after the previous refinement step, since even after refinement we typically have more than 1000 pairs of keypoint matches between frames. Thus, we find a consistent single transformation using the well-known “RANdom SAmple Consensus (RANSAC)” algorithm^42^. RANSAC iteratively estimates models from randomly chosen subsets of the set of observations and then chooses the model that generalizes best to the whole set. In each iteration, an affine transformation between consecutive frames is estimated using a randomly chosen subset of three matches and an error measure is calculated by generalizing this transformation to the rest of the matches. We then chose the affine transformation that minimizes the generalization error over the random subsets. In our experiments we observed that 3000 trials gave a good balance between computational complexity and generalizability. The list of matches is then pruned again by discarding keypoints whose displacement cannot be estimated with at least 50 pixel (*ℓ*_1_ distance based threshold that is determined experimentally) accuracy by the chosen transformation matrix.

We then run a final optimization over all matches that remain in this refined set and find the best transformation that describes their motion. The cost that we minimize is the sum of the *ℓ*_2_ distances between the estimated location of the keypoint matches in the latter frame and their real locations. Since we are matching consecutive frames that were collected through smooth motion of the microscope, the scale and warping introduced through the final registration transformation should be relatively small. However, when the set of matches remaining after RANSAC pruning happen to be spatially concentrated in a relatively small spatial portion of the frames, the transformation matrix is susceptible to over-fitting to that region, leading to distortions in the rest of the frame. In order to avoid such distortions, we added an additional penalty term to the cost that penalizes scale change and shear in the final registration, and thus avoids distortions due to over-fitting. The effect of this new penalty parameter is minimal as long as the matches are relatively homogeneously spread across the frames.

### Scene Cut Identification

To avoid distortion of the mosaic due to artifacts such as warping/distortion in frames or abrupt motion between them (Fig. 5), we cut the image sequence when an artifact is detected and start a new sequence from the subsequent frame. We automatically determine where to locate the cuts based on the calculated registration transformations. In particular, the diagonal coefficients of the transformation matrix control the amount of warping and distortion. Since a pure translation would result in those coefficients all being equal to 1, we place cuts when the trace of the matrix,ideally equal to 3, is above an experimentally determined threshold (in the work presented here the threshold used was 3.5). Once the cut locations are determined, each resulting subsequence is mosaicked separately. These subsequences can then be assembled manually or semi-automatically, and a mosaic covering the whole imaging area can be obtained. An example of this is shown in Fig. 1 (scene cut locations are shown by white arrows).

### Graph-Cut Based Stitching

After registration, given the large overlap between consecutive frames, we need to “merge” frames to construct the mosaic. As noted above, typical blending methods such as averaging or multi-resolution pyramid blending will lead to blurring, while the common alternative of choosing the maximum (or minimum) pixel value across the overlapped frames is not appropriate here since there is no reason to prefer dark versus bright intensities. In addition, the highly textured nature of RCM images will cause a loss of coherent structure and important cellular detail if extremal methods are used. Thus, we needed a method that keeps the actual pixel values while maintaining image fidelity. Simply choosing acquisition frame boundaries will lead to artifacts as well, due to imaging aberrations at frame edges as well as illumination changes between frames. Thus we devised an approach that meets this criteria by objectively choosing a single continuous boundary through the overlap area, driven by the pixel values themselves.

**Figure 6:**
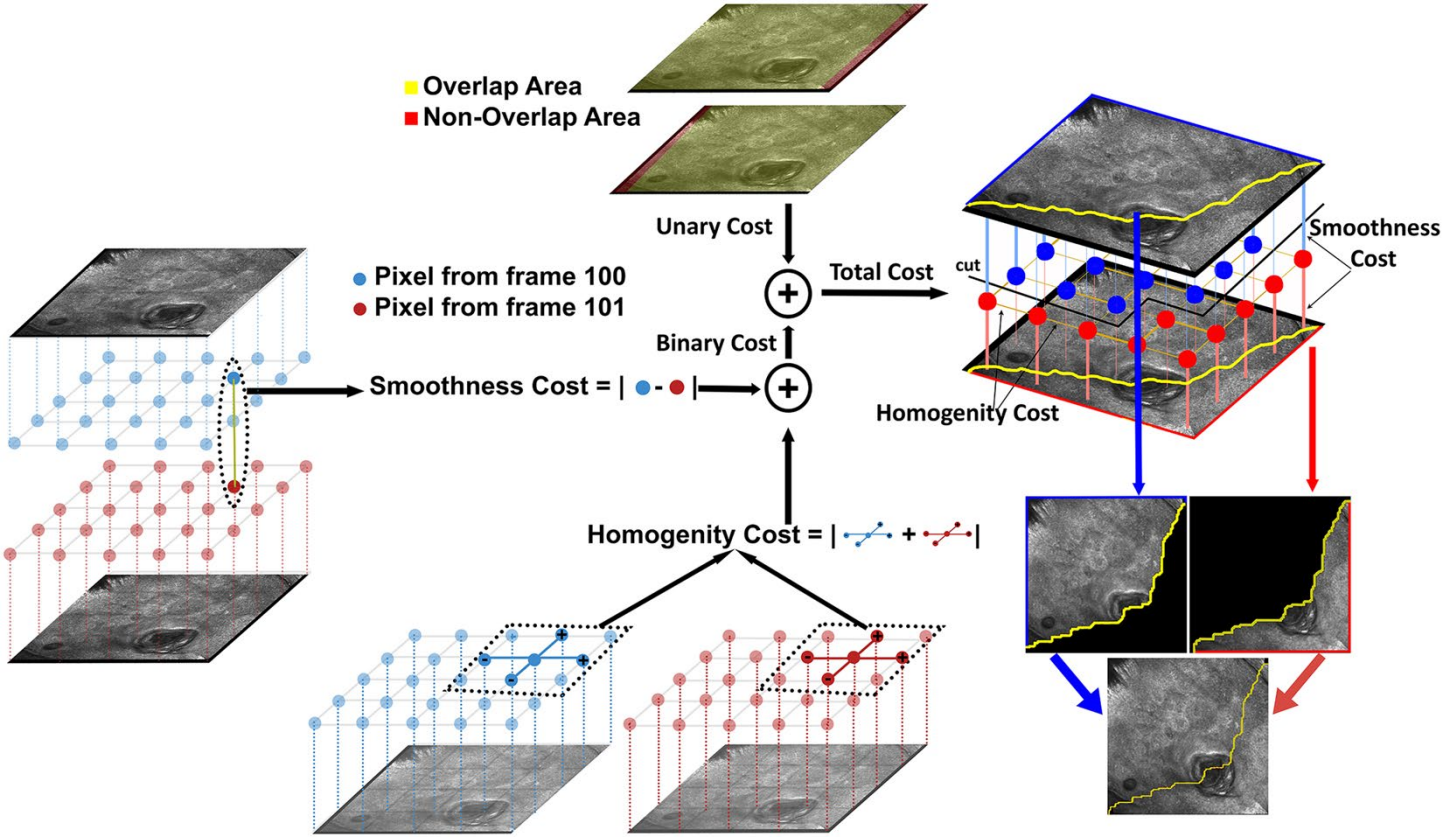
Example illustration of graph-cuts based stitching. In the overlapping areas of the registered images, the algorithm finds a stitching boundary by minimizing a local pixel variation based cost metric, which is composed of two parts a unary and binary cost. The unary costs (upper middle) depends on the intensity difference between overlapping images. The binary costs (lower middle and left) is calculated using intensity variations between neighboring pixel values within the images. The graph-cut algorithm finds a continuous stitching path (upper right) that goes through pixels in overlap area, whose accumulated cost is minimum among all possible such paths. The two images are then stitched together using this boundary (lower right).

For simplicity of exposition, we concentrate here on the case where only two frames overlap; the extension to multi-frame overlap is straightforward. Since we want to retain the original pixel intensities, we have two candidate pixels to choose between, one from the previous and one from the subsequent frame. Our stitching algorithm determines a contiguous boundary through the overlap region, with the mosaic on one side coming from the previous frame and on the other from the subsequent frame (Fig. 6). From a graph cut perspective the optimal boundary traverses the overlap region along the trajectory on which variation in the pixel values is minimum. To accomplish this, we assign a cost of having the stitching border go through each pixel, defined as the sum of (i) the absolute intensity difference between the candidate pixels from the two frames, and (ii) the absolute intensity differences between neighboring pixel locations in the same frame. We model the problem of estimating the optimal stitching border as a graph-cuts based labeling problem^33^, an approach which has been applied to many computer vision applications including photomontage^41^, texture synthesis^43^ and segmentation^44^, resulting in a data-driven optimized boundary. (For in-depth technical description of our implementation, please refer to supplementary materials).

## Electronic supplementary material

Supplementary Materials

LaTeX Supplementary File

LaTeX Supplementary File

LaTeX Supplementary File

LaTeX Supplementary File
