# Supplementary material for: Automated video-mosaicking approach for confocal microscopic imaging in vivo: an approach to address challenges in imaging living tissue and extend field of view
Source: Sci Rep. 2017 Sep 7;7:10759. doi: 10.1038/s41598-017-11072-9 (PMC5589933; doi:10.1038/s41598-017-11072-9)
Supplement: Supplementary file 1 — Supplementary Materials [file 41598_2017_11072_MOESM1_ESM.pdf]

Automated video-mosaicking approach for confocal microscopic imaging in vivo: an approach to address challenges in imaging living tissue and extend field-of-view

Kivanc Kose[1,+,*], Mengran Gou[2,+], Oriol Yélamos[1,3], Miguel Cordova[1], Anthony Rossi[1], Kishwer Nehal[1], Eileen Flores[1], Octavia Camps[2], Jennifer Dy[2], Dana H. Brooks[2], and Milind Rajadhyaksha[1]

[1]Memorial Sloan Kettering Cancer Center, Dermatology Service, New York, NY, USA
[2]Electrical and Computer Engineering, Northeastern University, Boston, MA, USA
[3]Dermatology Department, Hospital Clínic, Universitat de Barcelona, Barcelona, Spain
[*]kosek@mskcc.org
[+]these authors contributed equally to this work

Supplementary Material

In this section, we give further technical details of the methods that we developed or tailored for our developing our videomosaicking framework. For the sake of completeness, we briefly mention the standard methods that we used in the framework and refer readers to the cited literature for details. The focus of this supplementary material is on the modifications and additions that we made at individual steps of the framework. We will also provide the values of the parameters that were used during the implementation.

Extraction of SIFT Keypoints

In order to extract and describe keypoints, we used an off-the-shelf version of the SIFT algorithm[1] that was implemented in opencv[2] and ported into Matlab as mex libraries[3]. The SIFT algorithm has been extensively tested on natural images, from which values of its default parameters have been set. We made a few modifications in its default values to increase the efficiency of the algorithm for our specific reflectance confocal microscopy (RCM) data. SIFT is basically composed of 2 steps; detection of keypoints and calculation of the keypoint descriptors. In the SIFT algorithm, keypoints are defined by their high contrast relative to their neighbors. Useful keypoints may occur at different spatial scales (fine to coarse) of the image. SIFT explores this space using a Difference-of-Gaussian (DoG) representation in a two-step algorithm. First the image is downsampled repeatedly at different octave-based scales (e.g. downsampled by 2 in each direction). Then at each octave the image is filtered by Gaussian filters with different variances, and these images are then subtracted from each other. This procedure allows a search for keypoints at multiple octaves and at multiple scales within the octave, as illustrated in Figure S1. In order to obtain enough keypoints that lead to reliable matches between RCM frames, we experimented with different numbers of octave and scale parameters in the DoG representation (Figure S1). We observed that using a DoG representation with five octaves (corresponds to five times downsampling) and five scales (different variances of the Gaussian filter) at each octave gave a good balance between computational complexity and the number of extracted keypoints (which was typically ~ 3000 as noted in Methods). The initial variance of the Gaussian filters was $\sigma = 1.6$ pixels, and it increases as $k\sqrt(2)\sigma$ for $k \in [1, \ldots, 5]$

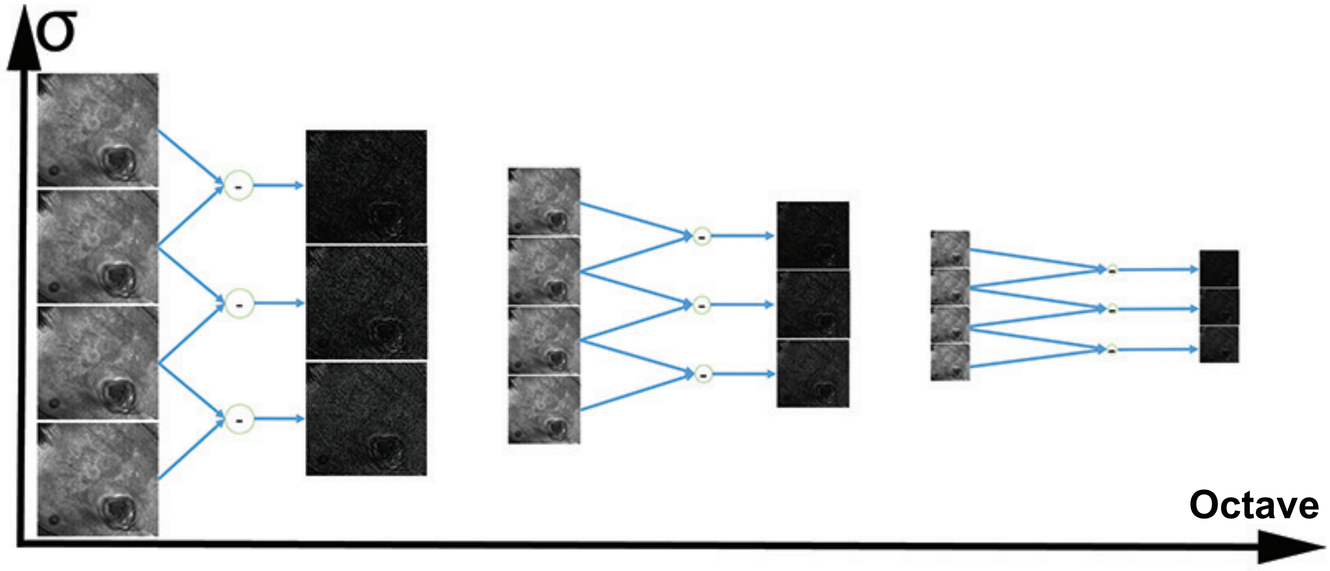

Figure S1. An example DoG representation: At each octave, the frame is convolved with Gaussian filters with increasing variances (called scales) and the convolved images are subtracted from each other to create the DoG representation at that particular octave. For illustrative purposes we show three DOG levels at three octaves, different from what we actually used in practice.

The second stage of SIFT is to calculate descriptors for each keypoint. We used standard SIFT descriptor[1]. These SIFT descriptors are feature vectors which concatenate histograms of the gradients at different angles in a spatial region around the keypoint. See Fig. S2 for a detailed illustration of this process.

Registration

Registration parameters between two consecutive frames can be calculated by analyzing the displacement of the keypoints in these images (optical flow). First, we matched the keypoints between two consecutive frames. The initial tentative matching is done by calculating a similarity metric between keypoint descriptors using an ℓ_2 (euclidean) distance. Each keypoint in each

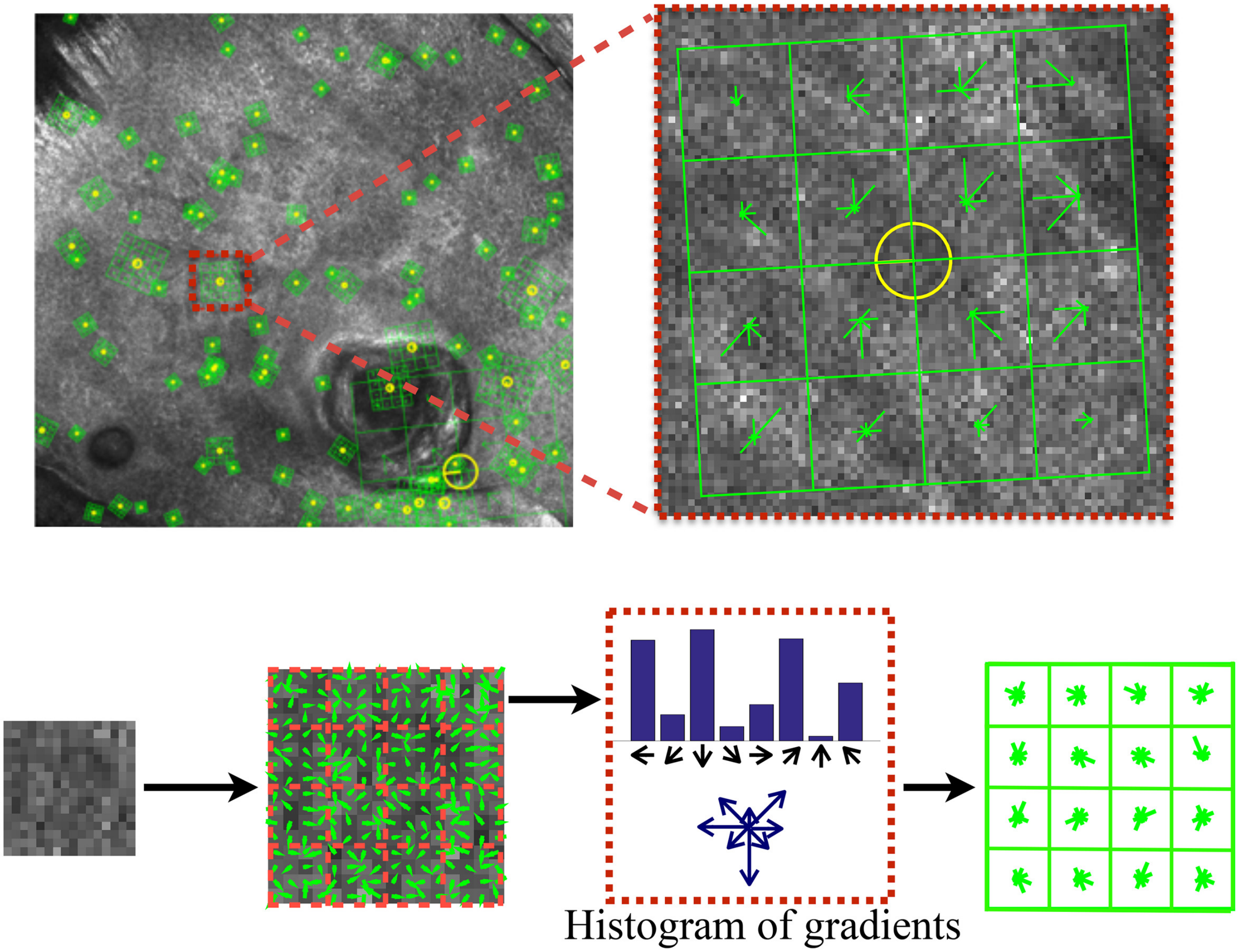

Figure S2. Histograms of gradients around each keypoint are used as descriptors. For each of the 16 subregions (each subregion is 4×4 pixels) around the keypoint, an 8 bin histogram of gradients is formed. These 16 histograms are concatenated to form a length-128 SIFT descriptor. For visual purposes, we only show a few of the keypoints

frame was matched with the 2 most similar keypoints in the following frame. Keypoints for which the ratio of its distance to its second closest match to its distance to the closest match was larger than 1.5 were counted as strong matches. The rest of the pairs (and the respective keypoints) were eliminated from the list of potential matches for subsequent processing.

Unfortunately, matching the keypoint based only on similarity between their descriptors is unreliable, as microscopic images often contain repeated textural patterns with similar appearance. For any microscope motion, all keypoints should move in a tandem fashion. Therefore, the relative displacement between the matched keypoints pairs must be similar, and thus should be well approximated by a single transformation matrix:

$$K_{t+1} = H_t K_t \tag{1}$$

where H_t is a 3-by-3 transformation matrix and K_t, K_{t+1} are triplets of homogeneous coordinates of matched keypoints between consecutive frames t and $t+1$. In general, for more than 3 matches, solutions to (1) will be inconsistent as we have many more constraints than free parameters. To robustly find a representative transformation, we used a procedure called RANSAC[4] to find an H_t that describes the motion between all matched keypoints with small error. In a single iteration, RANSAC solves (1) for one randomly-chosen triplet of matches, resulting in an estimate $\hat{H}_t^j$, where j is the iteration number. The resulting $\hat{H}_t^j$ is then used to calculate expected locations of all remaining keypoints from the earlier frame in the next frame. An estimation error E^j associated with transformation matrix $\hat{H}_t^j$ is then calculated by summing the ℓ_2 distance between the estimated keypoint locations ($H_t^j K_t$) and their actual location (K_{t+1}) in the following frame.

By solving (1) multiple times on randomly selected triplets, RANSAC calculates a vector of error values E^j, whose ℓ_2 is taken as measure of the robustness of H_t^j. The $\hat{H}_t^j$ matrix that results in the smallest error over all iterations is chosen as the transformation matrix $\hat{H}_t$. Testing many subsets is computationally costly, while testing on a smaller number of subsets leads to poor generalizability and thus poor registration. We observed experimentally that for the RCM images 3000 trials gave a good balance between computational complexity and generalizability.

Even with this choice of H_t, there are typically keypoint matches that are significantly inconsistent with the resulting transformation. Thus we made a second pass through the keypoint matches to discard those matches whose projected location in the second frame was not consistent with their actual location. Our experiments on RCM images suggested that discarding matches for which this location error was larger than 50 pixels gave a good trade-off between consistency and having too few matches. We will refer in the sequel to the remaining set of matches as inlier matches.

After RANSAC, the final step in registration is the refinement of $\hat{H}_t$ to best fit all inlier matches. In addition, in our application, we are only interested in registering consecutive frames that were collected during smooth motion of the microscope. In such cases the change in scale and warping (distortion) between the registered frames should be relatively small. Moreover, when the inlier matches are mostly concentrated on a portion of the frames, we want to prevent having a transformation matrix that is overfit to that portion of the image, which can lead to distortion in the rest of the frame. These goals can be characterized by an optimization problem which aims to find the registration transformation H_t that minimizes the total mismatch over all inlier matches while leading to the small frame-to-frame warping and scale change.

The total mismatch between the inlier matches can be calculated using the ℓ_2 error metric described before. The distortion induced by the registration can be quantified by analyzing the coefficients of the affine transformation matrix H. More specifically, the upper left 2-by-2 portion of the matrix controls the rotation and shearing in the registration and the entries in the first two rows of the last column (H(1,3) and H(2,3)) control the translation. Finally, the last row (H(3,1),H(3,2),H(3,3)) controls the projective geometry, which represents how the matches deviate from a 2D geometry. In order to avoid transformation matrices that lead to too much distortion, we introduced an additional cost parameter that penalizes the scale change and shear in the final transformation matrix. This parameter serves as a regularization term to force the final affine transformation matrix to favor translation and rotation motion between frames and limit shearing that may warp and distort the frame. Given the inlier matches $\bar{K}_t$ and $\bar{K}_{t+1}$ that we found through RANSAC, we solve the following optimization problem

$$min_H ||\bar{K}_{t+1} - H_t\bar{K}_t|| * (1 + pen), \quad (2)$$

$$pen = |1 - H_t(1,1)| + |1 - H_t(2,2)| + |H_t(1,2) + H_t(2,1)| + |H_t(3,1) + H_t(3,2)| * 10^2. \quad (3)$$

The first two terms in the penalty limit scale changes, the third term limits shearing and warping, and the last term limits the projective warping (deviation from 2D). Here we optimize Eq. 3 over all inlier matches. As an aside, we noted that, as expected, the effect of the additional penalty parameter was minimal when the inlier matches were homogeneously spread across the frames.

Scene Cut Identification

As described in Methods, our framework was designed to automatically detect sudden and unwanted motion of the microscope and then cut the longer sequence into internally consistent subsequences as needed. We developed a method that uses the coefficients of the transformation matrix H_t to determine where to cut. Since, as described in detail above in the Frame Registration section of the manuscript, the diagonal coefficients of H_t encode how much warping and distortion is introduced in the final registration, we quantify warping and distortion as

$$DeformationFactor = (1 - \frac{H(1,1)}{H(3,3)}) + (1 - \frac{H(2,2)}{H(3,3)}), \quad (4)$$

which is similar to the regularization term in Eq. (3). This factor quantitatively determines the degree to which the final transformation matrix imposes translation, rotation, and shearing. We place cuts between frames when this deformation factor is larger than 1. In addition, we calculate the ratio between the area covered by the earlier frame before and after the registration transformation. If this ratio is larger (smaller) than an experimentally determined threshold of 2.5 (1/2.5), then we also place a scene cut between those two frames.

Graph-cut based Stitching

As noted in the Methods section, in order to preserve resolution and cellular detail, we designed a method that retains the actual measured pixel values while ensuring image fidelity. Specifically, we adopted a graph-cuts based stitching algorithm that determines where to place a flexible, data-driven boundary that in effect composes the stitched image from two parts, each coming from pairs of neighboring original frames.

In the exposition here, we refer to the current state of the mosaic, composed from the previous and current images in the video sequence, as M and an ”incoming” frame that we wish to stitch as F. After registration we obtain a transformed version of F, which we denote F', which overlaps with some pixels of M. Thus in each location of the overlap area, we have two candidates pixels to choose from, one from M and the other from F'. The stitching step seeks a *single continuous stitching boundary* going through the overlap area, one side of which comes from M and the other side from F', as shown in Fig. 6 in the manuscript. We model the problem of finding the optimal stitching border as a graph-cuts based labeling problem, where the label $L(p)$ of each pixel p in the new video-mosaic is either M or F'. This pixel labeling problem can be approached as a graph partitioning by representing the pixels of the two images as the nodes of the graph. In this graph topology, labeling two neighboring pixels with different labels means the stitching boundary cuts the edge between these two pixels. The total cost of choosing a certain stitching boundary is associated with sum of the cost of the individual edges that the boundary cuts through (Fig. 6).

In our implementation the total cost of cutting an edge is composed of 2 terms as

$$C(L) = C_u(p, L(p)) + C_b(p, L(p)), \tag{5}$$

where the terms are the “unary cost” (C_u) and the “binary” cost (C_b)). Since we want to insure that the stitching boundary goes through the overlap area, we defined the unary cost as

$$C_u(p, L(p) = \begin{cases} 0 & p \in \{M \cap F'\} \\ \Omega & else \end{cases} \tag{6}$$

where $\{M \cap F'\}$ is the set of pixels in the overlap area of M and F' and Ω is a very large cost value, which always dominates the binary cost.

In the overlap region, the unary cost is set to be zero as noted above for all pixels because each pixel has the same equal probability of being in the final mosaic. Therefore, only the binary cost determines where the stitching border goes within the overlap area. The binary cost term for the cutting the connection between two pixels is calculated in two terms as:

$$C_b(p, L(p)) = C_s + C_h \tag{7}$$

where C_s, is the “similarity” cost, and C_h is the homogeneity cost. The similarity cost

$$C_s(p) = G_h(|M - F'|) \times K_h(p) + G_v(|M - F'|) \times K_v(p) \tag{8}$$

with

$$K_h(p) = \begin{cases} 0 & \text{if } L(p) = L(q),\ q \text{ is 2 horizontal neighborhoods of } p \\ 1 & \text{else.} \end{cases} \tag{9}$$

and

$$K_v(p) = \begin{cases} 0 & \text{if } L(p) = L(q),\ q \text{ is 2 vertical neighborhoods of } p \\ 1 & \text{else.} \end{cases} \tag{10}$$

is the absolute difference between the two candidate pixel intensity values from M and F'. It forces the stitching boundary to go through pixels with are similar in both M and F'. The homogeneity cost

$$C_h(p) = |\nabla_x M + \nabla_x F'| \times K_h(p) + |\nabla_y M + \nabla_y F'| \times K_v(p) \tag{11}$$

is the absolute intensity differences between candidate pixels and their spatial neighbors (“homogeneity” cost, C_h) (Fig. 6-Left column). It forces the stitching boundary to go through areas with small intensity variation.

Once the cost (function of labels) is calculated for each pixel, we minimize the overall cost with respect to the labels using Boykov’s graph-cut method[5]. In the highly unlikely case that this solution is not unique, the algorithm randomly picks one of the candidate stitching borders. In this way, the pixels of the final mosaic are uniquely chosen from pixels of either of the frames as shown in Fig. 6.

References

1. Lowe, D. Object recognition from local scale-invariant features. In *Computer Vision, 1999. The Proceedings of the Seventh IEEE International Conference on*, vol. 2, 1150–1157 (1999).

2. Bradski, G. *Dr. Dobb's Journal of Software Tools* (2000).

3. mexopencv. https://github.com/kyamagu/mexopencv. Accessed: 2016-11-27.

4. Fischler, M. A. & Bolles, R. C. Random sample consensus: A paradigm for model fitting with applications to image analysis and automated cartography. *Commun. ACM* **24**, 381–395 (1981). URL http://doi.acm.org/10.1145/358669.358692.

5. Boykov, Y., Veksler, O. & Zabih, R. Fast approximate energy minimization via graph cuts. *Pattern Analysis and Machine Intelligence, IEEE Transactions on* **23**, 1222–1239 (2001).
